# Sex Differences in the Neuropeptide Y System and Implications for Stress Related Disorders

**DOI:** 10.3390/biom10091248

**Published:** 2020-08-27

**Authors:** Roxanna J. Nahvi, Esther L. Sabban

**Affiliations:** Department of Biochemistry and Molecular Biology, New York Medical College, Valhalla, NY 10595, USA; rnahvi@student.nymc.edu

**Keywords:** females, neuropeptide Y, NPY receptors, stress, depression, age, early life stress, intranasal delivery

## Abstract

The neuropeptide Y (NPY) system is emerging as a promising therapeutic target for neuropsychiatric disorders by intranasal delivery to the brain. However, the vast majority of underlying research has been performed with males despite females being twice as susceptible to many stress-triggered disorders such as posttraumatic stress disorder, depression, anorexia nervosa, and anxiety disorders. Here, we review sex differences in the NPY system in basal and stressed conditions and how it relates to varied susceptibility to stress-related disorders. The majority of studies demonstrate that NPY expression in many brain areas under basal, unstressed conditions is lower in females than in males. This could put them at a disadvantage in dealing with stress. Knock out animals and Flinders genetic models show that NPY is important for attenuating depression in both sexes, while its effects on anxiety appear more pronounced in males. In females, NPY expression after exposure to stress may depend on age, timing, and nature and duration of the stressors and may be especially pronounced in the catecholaminergic systems. Furthermore, alterations in NPY receptor expression and affinity may contribute to the sex differences in the NPY system. Overall, the review highlights the important role of NPY and sex differences in manifestation of neuropsychiatric disorders.

## 1. Introduction

Sex plays a striking role in the development of stress-triggered disorders. Traumatic stress is more likely to lead to substance abuse in men [1,2] and prenatal stress in particular increases the risk of other psychiatric illnesses like autism spectrum disorder, attention deficit hyperactivity disorder, and schizophrenia in males [3]. In contrast, females have approximately double the susceptibility than males for many other stressed-related neuropsychiatric disorders like posttraumatic stress disorder (PTSD), depression, anxiety, and anorexia nervosa [4,5]. Current pharmacological treatment for stress-related neuropsychiatric disorders includes selective serotonin reuptake inhibitors (SSRIs), which take weeks to elicit an effect and reduce symptom severity rather than leading to remission of the disorders [6,7,8,9].

The NPY system is emerging as an important therapeutic target for preventing or reducing the incidence of neuropsychiatric disorders such as PTSD and depression. Recent studies demonstrate exciting potential of intranasal NPY or selective agonists to attenuate the development of immune-challenge and stress-elicited behavioral changes, as well as neurodegenerative diseases like Huntington’s disease [10,11,12,13]. Thus, it is imperative to further investigate possible causes of the sex specific differences in susceptibility to stress-related neuropsychiatric illnesses and its relationship to the NPY system.

The involvement of NPY in attenuating effects of stress, which has been studied primarily in males, has been the topic of a number of excellent, recent reviews [14,15,16,17,18,19,20,21,22,23]. However much less is known about NPY’s stress-responsive role in females. The information is scattered throughout the literature and thus is easily overlooked. Therefore, it is important to bring together information regarding sex differences in expression of NPY and its receptors. For the purposes of this review, we will focus on the sex differences in the NPY system in relation to stress disorders and its implications on the heightened susceptibility to stress in females. 

## 2. Stress-Protective Effects of NPY

NPY is a 36-amino-acid widely-conserved C-terminal amidated neuropeptide, one of the most prevalent peptides in the body, and has far-reaching effects both in the central nervous system and in the periphery. NPY is synthesized and processed from a larger precursor. In the brain, there is particularly high NPY expression in the hypothalamus, septum, nucleus accumbens, periaqueductal gray, and locus coeruleus. The amygdala, hippocampus, cerebral cortex, basal ganglia, and the thalamus also have abundant NPY [24]. NPY is involved in regulating a variety of physiological functions including feeding, circadian rhythm, anxiety, memory, fear, and stress [16,20,25,26]. It is thought that the stress-protective effects of NPY occur through modulating neurotransmission in stress-related brain regions, largely through inhibition of synaptic firing [22]. 

In the periphery, NPY affects the cardiovascular and immune systems, and also contributes to metabolic and skeletal homeostasis. It is localized with norepinephrine in neurons of the sympathetic nervous system [27]. Upon release, NPY acts as a vasoconstrictor and enhances the noradrenergic system’s response to stress [28]. NPY is also involved bone remodeling and promotes lipid accrual [29]. Emerging evidence suggests NPY also functions as an immunomodulatory agent. NPY treatment in a macrophage cell line increases levels of the anti-inflammatory cytokine TGFβ [30] and decreases IL-1β-induced pro-inflammatory effects [31,32,33].

**Figure 1:**
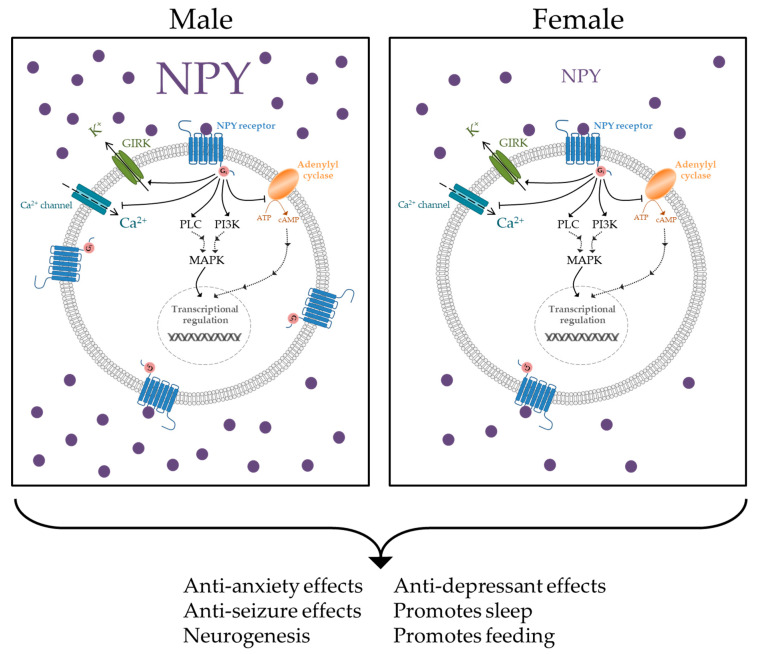
Neuropeptide Y (NPY) downstream signaling effects include inactivation of adenylyl cyclase and calcium channels, as well as activation of G protein-coupled inwardly-rectifying potassium (GIRK) channels, phospholipase C (PLC), and phosphoinositide 3-kinase (PI3K) [22,25]. The NPY system promotes numerous beneficial functions [57,58,59,60,61,62,63]. Females demonstrate less NPY peptide and Y1R expression as males in different brain regions, which could result in depressed signaling within the NPY system and a reduction in these beneficial effects.

There are five known NPY receptors (Y1R, Y2R, Y4R, Y5R, and y6R). Although y6R is active in rabbits and mice, it is non-functional in humans [25,34]. All NPY receptors are coupled to G_i/0_, which inhibits adenylyl cyclase and leads to decreased cAMP levels in the cell (Figure 1) [35]. Other downstream pathways affected by NPY include activation of MAPK, regulation of intracellular Ca^2+^ levels, and activation of G-protein-coupled, inwardly rectifying potassium (GIRK) channels, which serves to hyperpolarize the cell and reduce synaptic firing [22,36,37]. Selective agonist or antagonists of the NPY receptor subtypes have been shown to modulate behaviors associated with stress-disorders. For example, selective agonism of Y1R has been reported to mediate the anxiolytic and anti-depressive effects of NPY [38,39,40]. Whereas, Y1R and Y2R appear to regulate responses to conditioned fear [41]. 

In humans, studies related NPY with resilience to stress and lower levels of it with stress-related disorders like PTSD. Males with combat-related PTSD had lower levels of NPY in plasma and the cerebrospinal fluid (CSF) than combat-exposed controls [42,43,44]. Additionally, NPY levels in the CSF were lower in patients with reactive and first-episode depression [45,46], and its plasma levels were lower in patients with recurrent major depression as compared to controls [47]. Furthermore, NPY gene polymorphisms have been linked with stress-related behaviors [48,49,50].

Animal studies corroborate NPY’s stress-protective effects. Thorsell and colleagues demonstrated that overexpression of NPY conferred resilience to anxiety after restraint stress [51]. The intracerebroventricular (ICV) administration of NPY demonstrated anxiolytic effects and improved fear extinction [52,53,54]. When NPY is genetically knocked out, animals demonstrate accelerated fear acquisition and delayed fear extinction [41]. NPY has also been reported to attenuate stress-triggered elevation of norepinephrine biosynthetic enzymes, as well as the hypothalamus–pituitary–adrenal (HPA) axis [55,56].

Extensive literature indicates that there are sex differences in the NPY system in relation to stress within both the central nervous system and periphery [64,65,66,67,68,69,70,71,72]. In order to better understand its role in different biological processes, it is imperative to investigate NPY’s actions in a sex-specific manner. Increased knowledge of sex differences within the NPY system could translate to improved health benefits in terms of greater understanding of disease progression and application of therapeutic interventions.

## 3. Sex Differences in NPY Expression in Unstressed Conditions

Sex differences in NPY expression levels were determined in unstressed conditions within several stress-related brain regions in rodents (Table 1). Overall, females have been found to have lower NPY levels and less NPY-expressing cells than males. Females have lower NPY peptide levels than males in the hypothalamus, a brain region involved in the canonical HPA axis [64]. Studies on specific hypothalamic nuclei demonstrate a similar pattern. The arcuate nucleus of the hypothalamus is involved in maintaining energy homeostasis and is one of the major sources of NPY in the brain [73,74]. Additionally, the arcuate nucleus is a site of negative feedback for the HPA axis [75]. Projections from NPY neurons of the arcuate nucleus to corticotropin-releasing hormone (CRH) neurons in the paraventricular nucleus of the hypothalamus could contribute to negative regulation of the HPA axis [76]. Sex differences in arcuate nucleus NPY expression thus would have consequences in regulation of the HPA axis and stress responses. Urban and colleagues showed that female rats exhibited less NPY mRNA-containing cells in the rostral-caudal portion of the arcuate nucleus than males [71]. Lower NPY gene expression levels in the arcuate nucleus suggest that homeostatic functions, including responses to stress, may be better regulated by NPY in males than females. 

Just inferior to the arcuate nucleus is the median eminence, the portion of the hypothalamus from where regulatory hormones such as CRH are released [77]. Upon stress, the median eminence rapidly discharges CRH into the surrounding environment and the hypophyseal portal circulation [78]. CRH then acts on the anterior pituitary gland through the hypophyseal portal circulation to release ACTH, which travels to the adrenal gland to stimulate the release of corticosterone directly [79,80] and catecholamines, possibly indirectly [81,82,83]. NPY was shown to inhibit ACTH and cortisol release [84]. In females there are approximately 50% lower NPY peptide levels in the median eminence as compared to males [70]. The sexually dimorphic NPY levels in the median eminence might indicate that females are likely less able to negatively regulate secretion of regulatory hormones, including ACTH. 

The lower expression of NPY in these specific hypothalamic nuclei could contribute to the increased activation of the HPA axis, resulting in higher susceptibility of stress-related disorders in females. Indeed females have higher plasma corticosterone after stress exposure [85,86]. Although many studies report females expressing less NPY than males in the hypothalamus, one study found females had higher levels of NPY in the hypothalamus [65]. However in this study, the animals were fasting prior to NPY measurement, which has been demonstrated to increase NPY expression in the hypothalamus [87,88,89] with important sex differences in response to stress [90,91]. 

Additionally, sexually dimorphic NPY levels have been reported in other brain regions, such as the striatum and hippocampus. Females have less NPY in the striatum than males [64]. The striatum is involved in the reward circuitry and has implications in stress-related disorders. Individuals with PTSD demonstrate impaired striatal activation and have decreased striatal gray matter [92,93,94]. In the striatum, NPY increases the release of dopamine, a neurotransmitter famously involved in reward [95,96]. Additionally, NPY is neuroprotective against cellular stresses in the striatum [97]. These findings suggest that higher levels of NPY in the striatum abate the related stress-induced impairments. Therefore, the sex differences seen in striatal NPY levels might contribute to the higher prevalence of stress-related disorders in females. 

The hippocampus is largely known for its involvement in learning and memory; however, it also functions in stress-related impairments such as anxiety and conditioned fear [98,99]. Individuals with PTSD have decreased hippocampal volume and activity, speculated to be as a result of over-excitation after experiencing traumatic events [100,101]. In addition to its neuroproliferative effects, NPY decreases many stress-related receptors and molecular effectors in the hippocampus, including the glucocorticoid receptor [12,55,58]. In unstressed conditions, hippocampal NPY levels were lower both in prepubescent and adult females as compared to males [64]. These sex differences in the hippocampus could have large implications in the female response to stress. Activation of the limbic circuitry of the hippocampus causes anxiogenic behaviors, in part due to its encoding of emotional memories and input from the amygdala [102,103]. Overexpression or microinfusion of NPY into the hippocampus of male rodents confers resilience to anxiety and conditioned fear after stress [51,104]. Thus, lower basal hippocampal levels of NPY in females could contribute to their increased susceptibility to anxiety disorders. 

Another key brain region involved in the stress response is the locus coeruleus. It is the main source of norepinephrine for the central nervous system and plays a key role in many symptoms seen in stress disorders like PTSD, such as hyperarousal, impaired cognition, anxiety etc. [105,106,107,108,109]. NPY decreases the release of norepinephrine from the locus coeruleus [110,111]. Furthermore, NPY infusion into the region of the locus coeruleus in male rats produced anxiolytic behavior [112]. After exposure to stress, intranasal NPY administration in males attenuates the induction of tyrosine hydroxylase, the rate-limiting enzyme for norepinephrine, in the locus coeruleus [12]. Sex differences in NPY expression within the locus coeruleus have not been investigated and could provide crucial insight into the increased susceptibility of stress-related disorders in females. This is especially important as the locus coeruleus demonstrates sexual dimorphism in the response to CRH [113,114,115,116] and women exhibit increased activation of the locus coeruleus than men in response to visual emotional stimuli [117].

Estrogen is known to play a regulatory role in NPY expression. Estrogen increases the number of NPY neurons, amount of NPY in pre-synaptic boutons and NPY release in the hippocampus [57,118]. Furthermore, estrogen increases NPY mRNA expression in the hippocampus and caudate nucleus [119]. Animals in the proestrus, or “high-estrogen”, phase of the estrus cycle have higher NPY mRNA levels in the arcuate nucleus than in the metestrus, “low-estrogen” phase [120]. Interestingly, intra-lateral septal infusions of NPY produced an anxiolytic effect at lower doses in proestrus animals, whereas only the highest doses produced an anxiolytic effect in metestrus-diestrus females [121]. It is conceivable that there is a threshold of NPY expression that needs to be met in order to produce a therapeutic behavioral effect. In this case, animals in proestrus that have higher NPY expression would be closer to meeting that threshold and require relatively less exogenous NPY to achieve a therapeutic benefit. Interestingly, even females in proestrus had less NPY mRNA-containing cells in the arcuate nucleus than males [71]. It is also important to note that NPY demonstrates a dose-response anxiolytic effect, where higher concentrations of NPY are therapeutic over lower concentrations [52,122]. Therefore, it is also important to consider the estrus cycle and fluctuating hormone levels when investigating the NPY system in females.

## 4. Sex Differences in NPY Knockout Animals and Stress-Related Genetic Models

Several studies have examined the effects of sex differences when NPY is knocked out (KO). Both male and female NPY KO mice exhibited increased depressive-like behavior on the forced swim test (FST) [62]. As compared to wildtype (WT) controls, NPY KO females did not differ in anxiety-like behavior on the elevated plus maze (EPM) or the light-dark anxiety tests, however they did exhibit anxiogenic behavior on the open field test. In contrast, NPY KO in males displayed greater anxiety-like behavior on all these measures as compared to WT controls [60,62]. These results from the KO studies suggest that NPY is involved in regulating depressive-like behaviors but plays a lesser role in basal anxiety in females. However, ICV administration of NPY into the lateral ventricle before testing on the EPM was anxiolytic in females [59], as well as in males [52]. The global deletion of NPY in the NPY KO mice could have triggered compensatory mechanisms specific to females pertaining to the regulation of anxiety behavior. It is possible that in females NPY plays a role in anxiety; however it may work in concert with other unknown, sex-specific mechanisms.

Another means of examining the biology of stress is to use genetic animal models reproducing the phenotypes associated with stress-related disorders. The Flinders Sensitive Line (FSL) rat has been used as an animal model for depression, whereas the Flinders Resistant Line (FRL) rat serves as its control. In both the FSL and FRL, females have lower NPY levels than males in the hippocampus. In both males and females, the FSL animals had less NPY peptide levels within the hippocampus than their FRL counterparts in both sexes [68], and female FSL rats had less hippocampal NPY mRNA levels than Sprague-Dawley controls [123]. Moreover, in female FSL rats, there was a negative correlation between hippocampal NPY mRNA levels and duration immobile on the FST, a measure of depressive-like behavior [124]. The behavioral correlate of FSL with depression and its sexual dimorphic NPY expression suggest that depressed and depression-resilient females have less NPY than their respective males. Furthermore, it suggests NPY levels are associated with resilience to depressive behaviors in both sexes. These findings lend themselves to the possibility that the higher incidence of depression in females could be due to differences in NPY expression.

## 5. Sex Differences in Brain-Specific and Peripheral NPY Expression after Stress

Sex differences in NPY expression are evident after exposure to stress (Table 2). However, the age of exposure may be an important factor in determining the effects of stress on NPY expression and its sex differences. Within the arcuate nucleus, early-life stress triggered a greater increase in NPY fiber density in females as compared to males when examined at 14 days postnatally [125]. In the amygdala, early-life stress increased NPY mRNA levels at 30 days postnatally in females, only [126]. When examined as adolescents, early-life stress decreased NPY neuron density in the arcuate nucleus equally in both sexes, as compared to unstressed controls [127]. However, when animals exposed to early-life stress were examined in adolescence, females tended to exhibit lower NPY expression in specific brain regions. Specifically, these females showed fewer NPY-immunoreactive neurons than males in both the basolateral amygdala and the hippocampus after exposure to maternal separation stress [66]. Furthermore, adult female rats had lower NPY peptide levels in the dorsal hippocampus after maternal separation stress than males [67]. Early-life stress caused a decrease in NPY-expressing neurons and peptide levels in both females and males as compared to unstressed controls [66,67,128]. Perhaps stress acutely hyperactivates the NPY system, but it is attenuated after time in females. It is important to note that the NPY measurements discussed above were taken from different brain regions. More studies examining NPY expression at different time points after stress within the same brain region are needed to further elucidate this issue. 

Aside from early-life stress, stress sustained in adulthood also impacts NPY expression levels in females. Chronic social isolation stress decreased hippocampal NPY mRNA expression in females [123]. Exposure of females to chronic variable mild stress increased NPY mRNA levels in the prefrontal cortex as compared to unstressed controls when examined 3 days after the last day of stress. This effect was not seen in males [129]. However, there have been other studies that demonstrate chronic variable stress has an effect in males. In one study, NPY peptide expression increased in the prefrontal cortex 7 days after exposure to the stressors in males as compared to unstressed controls [130]. It is possible that the time course after the stressor contributes to the sex differences in NPY expression even in adulthood. Additionally, the nature and duration of the stressors could play a factor in the differences observed.

In the periphery, NPY is colocalized with and acts to augment the vasoconstrictor effects of norepinephrine [27,28]. The sympathetic nervous system largely contributes to circulating levels of NPY and norepinephrine [131]. As discussed earlier, lower plasma levels of NPY have been associated with PTSD and major depressive disorder, both of which have a higher prevalence in females [4,5,42,47]. In unstressed conditions, females displayed lower plasma levels of NPY than males [65]. Twenty minutes after exposure to cold stress, only males had increased plasma NPY levels. Additionally, when males and females were compared after cold stress exposure, females had less circulating NPY than males [69,132]. When the plasma was examined immediately after exposure to cold stress, NPY levels increased in females as compared to unstressed controls [133]. In both males and females, circulating NPY levels increased after restraint stress. However, females overexpressing NPY in noradrenergic neurons demonstrated higher levels of NPY in plasma than their wildtype controls after stress. This effect was not seen in the respective male groups [133]. Furthermore, circulating NPY levels increased after cold stress in demedullated females, whereas no change was seen in demedullated males [69]. It is possible that control of the stress response, particularly with regards to NPY, is more dependent on the catecholaminergic system in females than males. It is worth investigating sex differences in the balance and interaction between NPY and catecholamines to better elucidate the depressed NPY system in females. 

Perhaps females are not able to sustain the same increase in circulating NPY levels as males because of decreased production or increased degradation. One study reported decreased basal and stimulated NPY secretion from cultured adrenal medulla in females relative to males in the presence of a NPY protease inhibitor [69]. Furthermore, when NPY protease was inhibited in the adrenal medulla, a greater increase in NPY levels was seen in males than in females [69]. This effect suggests that degradation occurs to a larger extent in males as compared to females. Moreover, when degradation is inhibited, females continued to have lower NPY levels than males, indicating that decreased synthesis or release may be a larger contributing factor to the lower circulating NPY levels seen in females.

**Table 2:** Sex differences in NPY expression levels after stress.

Tissue	Type of Stress	Time of Measurement	Results	Species	Reference
Hypothalamus: Arcuate nucleus	Limited nesting and bedding at PND 2–9	PND 14	Stressed 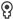 > stressed 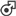 NPY fiber density Stressed > unstressed NPY fiber density in both 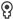 & 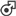	C57BL/6J mice	[125]
Hypothalamus: Arcuate nucleus	24 h maternal separation at PND 3 or 11	PND 53–60	Stressed < unstressed NPY neuron density in both 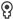 & 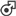 no sex differences were found	Wistar rats	[127]
Amygdala	Caregiver maltreatment 30 min daily from PND 1–7	PND 30	Stressed > unstressed NPY mRNA levels ( 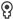 only)	Long-Evans rats	[126]
Basolateral amygdala	24 h maternal separation at PND 3 or 11	PND 52–60	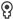 < 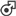 NPY immunoreactive cells in controls and both stressed groups	Wistar rats	[66]
Basolateral amygdala	Maternal separation 3 h daily from PND 3–14	PND 62	Stressed < unstressed NPY-immunoreactive cells ( 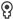 )	Wistar rats	[128]
Hippocampus	24 h maternal separation at PND 3 or 11	PND 52–60	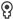 < 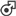 NPY immunoreactive cells in controls and both stressed groups	Wistar rats	[66]
Dorsal hippocampus	Maternal separation at PND 2–6 and 9–13 for 6 h/day	PND 84	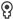 < 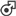 NPY peptide levels	Sprague-Dawley rats	[67]
Hippocampus	FSL	PND 77	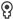 < 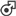 NPY peptide levels	Rats from FSL and FRL	[68]
Hippocampus	FSL only or FSL + social isolation stress for 7 weeks	Immediately after stress	FSL < SD rats NPY mRNA levels in both unstressed and stressed ( 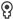 )	Sprague-Dawley rats & rats from FSL	[123]
Hippocampus	Social isolation stress for 7 weeks	Immediately after stress	Stressed < unstressed NPY mRNA levels ( 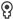 )	Sprague-Dawley rats	[123]
Prefrontal cortex	Chronic mild variable stress for 21 days	3 days after last stressor	Stressed > unstressed NPY mRNA levels ( 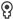 only)	C57BL/6 mice	[129]
Plasma	Cold stress		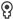 < 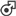 NPY peptide levels	Rats	[69]
Plasma	Restraint stress for 60 min	Immediately after stress	NPY OE-NA stress > WT stress ( 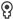 only)	C57BL/6 mice	[133]
Plasma	Intimate Partner Violence	4 months–2 years since time of abusive relationship	Childhood abuse negatively correlated with lower plasma NPY levels in IPV participants ( 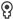 )	Humans	[134]
Plasma	Childhood Sexual Abuse	12–29 years since time of sexual abuse	CSA < controls NPY peptide levels ( 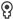 )	Humans	[135]

Abbreviations: post-natal day (PND); Flinders Sensitive Line (FSL); Flinders Resistant Line (FRL); Sprague-Dawley (SD) NPY over-expressed in noradrenergic neurons (OE-NA); Intimate Partner Violence (IPV); Childhood Sexual Abuse (CSA). 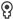/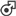: Female/Male.

## 6. Sex Differences in Brain-Related NPY Receptor Expression in Unstressed Conditions and after Stress

There are few studies on sex differences in NPY receptor levels (Table 3). One study reported lower NPY receptor subtype 1 (Y1R) density in adult female cortex than males. Interestingly, cortical Y1R had higher affinity for NPY in females as compared to males. When comparing the abundance of cortical NPY levels or G_i_α in these animals, there was no difference between the sexes [72]. The lower Y1R levels might be compensated with higher Y1R-NPY affinity in females. Another possible explanation is that Y1R plays a less important role in females as compared to males, since less of it is present at baseline.

Alternatively, less Y1R could contribute to susceptibility to the effects of stress in females. We found that mRNA levels of Y1R and CRH receptor subtype 1 (CRHR1) in the locus coeruleus increased in female rats when examined 1 week or more after exposure to single prolonged stress as compared to unstressed controls [136]. The increase in gene expression could be a way to respond to stress and, in the case of Y1R, ameliorate the downstream effects of CRHR1. If so, the availability of Y1R would be especially important in females as they have a hyper-reactive locus coeruleus in response to CRH as compared to males. This hyper-reactive locus coeruleus includes increased afferents receiving input from the limbic system and CRHR1 availability in females [114,116]. Furthermore, this elevation of Y1R gene expression levels was not seen in males, suggesting the potential importance of Y1R in females [56,137]. 

Estrogen plays a role in the regulation of Y1R within stress-related regions. Estrogen response elements flank the Y1R gene and its gene expression is upregulated by estrogen [138]. Y1R expression is increased in the hypothalamus during the high-estrogen phase of the estrus cycle (proestrus), as compared to the other phases of the estrous cycle [139,140]. Furthermore, exogenous estradiol benzoate administration to ovariectomized females increased hypothalamic Y1R gene expression, as compared to controls [140]. These studies further highlight the potential importance of Y1R in stress-related disorders in females. 

Knockout studies provide additional information on the role of Y1R on stress-related behaviors in females. Unstressed, Y1R KO females spent less time immobile on the tail suspension test compared to wildtypes (WT); however, they did not differ when examined on the force swim test (FST), both of which have been used to demonstrate predictive validity for antidepressant therapies and loosely used as measures of depressive-like behavior. Nonetheless, after stress, Y1R KO females spent less time immobile on the FST than WT controls. Furthermore, Y1R KO females had increased locomotor activity 1 week after forced swim stress [141]. This suggests that Y1R may contribute to depressive-like behavior and decreased locomotion in females. However, another study found that Y1R KO mice exhibited greater immobility on FST as compared to WT when males and females were analyzed as one cohort [142]. Furthermore, there was a positive correlation between hippocampal Y1R mRNA levels and swimming behavior on the FST in FSL females [124].

Anxiety behavior changes in Y1R KO animals were dependent on the diurnal cycle in males. There were differences in anxiety behavior in Y1R KO males as compared to WT controls when examined in the second half of the light cycle, but not the first [143]. Similarly, Y1R KO females did not differ from WT controls in anxiety measures when examined early in the light cycle, regardless of stress status [141]. Furthermore, a combined male/female cohort of Y1R KO mice had no differences in anxiety measures as compared to WT controls in unstressed conditions [142]. However, when the Y1R gene was inactivated in Y5R-expressing neurons, males and females exhibited increased anxiety [144]. Y5R functions to regulate anxiety along with Y1R and the two are colocalized in limbic system brain regions [52,145,146]. Thus, the selective Y1R inactivation from brain regions involved in emotion regulation provides a higher resolution of its functions in stress-related behaviors, as opposed to a global knockout. These findings provide further support that specific perturbation of the NPY system, and specifically Y1R, induces behavioral changes pertaining to stress-related disorders in females.

Y2R, located predominately presynaptically, negatively regulates the release of neurotransmitters from the presynaptic neuron, including norepinephrine, GABA, glutamate, and NPY [147,148,149,150,151]. Depending on which brain system or structure is affected, antagonism or deletion of Y2R could produce different results. When Y2R was knocked out in GABAergic neurons, both males and females had increased depressive-like behavior as measured by the FST [152]. However, the GABAergic neuron-specific Y2R KO increased sucrose preference as compared to WT controls in males only [152]. The relative increase in sucrose preference, an indicator of hedonic behavior or absence of anhedonia, reflects an improvement in depressive behavior in Y2R KO males. Male-specific changes within the Y2R system are also seen after exposure to stress. We observed a decrease in Y2R gene expression levels in the locus coeruleus after single prolonged stress as compared to unstressed controls only in males [56,136,137]. A decrease in Y2R expression would allow for more NPY and other neurotransmitters to be released into the synapse. It is possible that there was a compensatory decrease in Y2R expression as part of the stress-response that is male-specific.

The current literature suggests a sex-specific emphasis on the effects of different NPY-receptor subtypes. While it is likely that both Y1R and Y2R contribute to regulating the stress response in both sexes, it is possible that Y1R and Y2R in specific stress response brain regions play more significant roles in females and males, respectively. Further studies are needed to elucidate the sex-specific stress response mechanisms of the NPY-receptor subtypes within specific brain regions.

## 7. Sex Differences in Response to NPY Administration

Our laboratory has previously shown that intranasal administration of NPY to rats can prevent and reverse deleterious effects of traumatic stress in males subjected to the Single Prolonged Stress model of PTSD. Administration of intranasal NPY shortly before stress exposure (prophylaxis) prevented the development of depressive-like behavior, anxiety and hyperarousal. Additionally, it prevented activation of the HPA axis and the locus coeruleus-noradrenergic system [12]. Similar results were obtained when NPY was administered immediately after exposure to stress (early intervention) [12,55,56,153,154]. Intranasal NPY effectively reversed hyperarousal, depressive-like and anxiety behavior seen in vehicle-treated animals when it was given a week or more after exposure to stress [137,155]. Furthermore, intranasal NPY attenuated impaired locomotion and exploration in immune-challenged mice [11] and disease pathology in a mouse model of Huntington’s disease [10]. Thus, intranasal NPY shows promise as a putative effective treatment for stress-related disorders in males.

These encouraging results prompted us to examine the therapeutic potential of intranasal NPY as an early intervention in females. At the same doses effective in males, intranasal NPY did not prevent the development of depressive-like or anxiety behavior after exposure of females to stress. Similarly, stress-elicited molecular changes in the locus coeruleus were not altered [136]. 

Interestingly, a recent clinical trial tested the effects of intranasal NPY in a cohort of patients with PTSD. The group found a dose-response effect of intranasal NPY on the Beck Anxiety Inventory [122]. In this study, 67% of the participants were female. Furthermore, ICV administration of NPY and selective agonists at different doses demonstrated that higher concentrations were more anxiolytic than lower concentrations in male rats [52]. Perhaps there is a similar dose-response effect in females and with intranasal administration. While the sex differences in response to intranasal NPY administration after stress may be a result of lower NPY in females, the mechanism is still unclear. Further studies are needed to determine whether females are responsive to NPY at higher doses or if NPY is unable to attenuate stress responses in females.

## 8. Human Studies in Women and Age-Dependent NPY Expression Changes

Few studies have studied NPY levels in females as they relate to stress in a clinical setting. In one study, females inmates with a history of childhood sexual abuse had lower plasma NPY levels than those without a history of sexual abuse [135]. In another study, women with history of sexual abuse and psychogenic non-epileptic seizures had lower plasma NPY levels than healthy controls with and without abuse [156]. Seedat and colleagues examined plasma NPY levels in females who have had a history of intimate partner violence (IPV) as compared to others. There were no differences in plasma NPY levels between the two groups. However, when plasma NPY levels were compared with scores on the Childhood Trauma Questionnaire (CTQ), the study found a negative correlation. Specifically, IPV participants who had higher scores on the physical neglect subscale had lower plasma NPY levels [134]. This finding translates some of the preclinical early life stress studies discussed earlier. It is possible that NPY is closely linked to early life trauma in females. A limitation of these clinical studies is their peripheral measure of NPY.

Interestingly, there is a link between age and NPY levels specifically in females, as well as age of sustained trauma with the risk of developing PTSD. Several studies report an increase in NPY levels with age in healthy females. CSF levels of NPY-like immunoreactivity (NPY-LI) increased with age only in women, but not in men [157]. In the hypothalamus, NPY gene expression levels per neuron were elevated in older women (ages 59–86) as compared to younger women (ages 21–39) and were positively correlated with age [158]. Interestingly, ovariectomy did not change hypothalamic NPY gene expression as compared to intact females [158]. Furthermore, plasma levels of NPY-LI increased with age in healthy women [159] and postmenopausal women had higher plasma NPY levels than cycling females at all stages of the menstrual cycle [160]. Plasma levels of DPP-IV, one of the proteases for NPY and other peptides such as proinsulin, did not differ between postmenopausal women and cycling females [160], which suggests the increase in plasma NPY levels in postmenopausal women did not occur as a result of altered degradation of NPY. 

Overwhelming evidence in preclinical studies shows that NPY expression decreases with age in males, as opposed to the observed increase in females. Within the arcuate nucleus, aged males had less NPY gene expression than young animals [161,162]. Similarly, aged males had less NPY peptide expression within many hypothalamic nuclei than young rats [163,164,165]. Furthermore, there was less stimulated release of hypothalamic NPY in aged rats than young males [163]. These NPY expression changes have been linked with the decrease of testosterone with age in males [161,163,164].

The age-related differences of NPY levels could contribute to the age-dependent risk of developing PTSD in females. Females with childhood traumatic events were more susceptible to developing PTSD than those who experienced traumatic events in adolescence and early adulthood [166]. Among those with a history of child abuse and/or neglect, females were more likely to exhibit internalizing symptoms during adolescence [167] and were more than twice as likely as men to develop PTSD as adults [168]. Furthermore, the female to male ratio of PTSD prevalence was highest among adolescents and young adults at approximately 3:1 and decreased to approximately 2:1 among those ages 66 to 70 years old [4,169]. These age-dependent differences in NPY levels deepen our understanding of stress-response regulation in females and warrant further investigation. 

## 9. Future Directions

More studies assessing NPY and its receptor levels in specific brain regions associated with stress in females are needed to better understand NPY’s involvement in stress-related disorders in a female clinical population. Recent advances in development of PET tracers for neuroimaging of Y1R, Y2R and Y5R show promise for human studies [170,171,172,173]. Additionally, studies with a focus on time-specific measures in relation to the time of stress are important to establish a temporal relationship between trauma and NPY in females and influence of estrous/menstrual cycle. This includes examining the effects of stress on NPY and its receptor levels at various time points after the time of stress, as well as at what age the trauma occurred. Furthermore, it is critical that future studies investigating the clinical use of intranasal NPY examine sex differences in response to treatment. As demonstrated in preclinical studies, females do not respond to intranasal NPY treatment the same as males and may require higher doses to reach therapeutic effects. It is imperative to understand these differences as they can have a critical impact on treatment strategies. 

## 10. Conclusions

NPY is known to regulate many effectors of the stress response. A preponderance of literature indicates that NPY concentrations in the brain are lower in females than in males under both basal and stressed conditions. This suggests that either the depressed NPY system in females may be less important for stress regulation or may contribute to their higher prevalence of stress-related disorders. Early life stress in females appears to have a strong link with NPY levels in adulthood, and age plays a role in NPY expression in females, as well. Furthermore, there may be differential regulation of NPY receptors in relation to stress between females and males. Further investigation of these sex differences in the NPY system could shed new light on the mechanisms behind the heightened susceptibility of females to stress-triggered neuropsychiatric disorders and approaches to therapeutic interventions.

**Main Conclusions**

- Neuropeptide Y (NPY) has wide-reaching effects in the central nervous system and periphery, including stress-protective effects [16,20,25,27,28,29,30,31,32,33].
- Females have less NPY expression than males in unstressed conditions within many stress-related brain regions and in plasma [64,65,70,71], with the estrogen playing a potential role in its regulation [57,118,119,120].
- Genetic models show the NPY system is involved in regulating depressive-like behaviors in both sexes, while its effects on anxiety is more pronounced in males [60,62,68,123,124].
- The majority of studies indicate that females have less NPY expression in adulthood than males after early life stress in several brain structures involved in the stress response [66,67], as well as in plasma after exposure to cold stress [69].
- Females have lower Y1R membrane expression, but higher Y1R-NPY binding affinity than males in cortical tissue [72]. Estrogen contributes to the regulation of Y1R expression [138,139,140].
- Intranasal NPY administration may be an effective treatment for stress-elicited disorders in both sexes [122], however females may require a higher concentration as a result of lower endogenous NPY expression.
- Central and peripheral NPY expression increases with age in females [157,158,159,160], while it decreases with age in males [161,162,163,164,165].

## Figures and Tables

**Table 1:** Sex differences in NPY expression levels in unstressed conditions.

Tissue	Results	Species *	Reference	Additional Notes
Hypothalamus	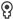 > 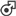 NPY peptide levels	C57BL/6 mice	[65]	
Hypothalamus	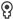 < 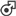 NPY peptide levels	Rat	[64]	
Hypothalamus: Rostral-caudal arcuate nucleus	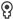 < 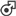 NPY mRNA-containing cells	Sprague-Dawley rats	[71]	
Hypothalamus: Median eminence	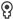 < 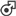 NPY peptide levels	Sprague-Dawley rats	[70]	
Striatum	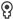 < 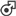 NPY peptide levels	Rat	[64]	
Hippocampus	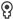 < 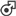 NPY peptide levels	Rat	[64]	Prepubescent and adult females had less NPY levels than respective males
Plasma	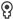 < 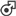 NPY peptide levels	C57BL/6 mice	[65]	

* The species were reported as specified in the original studies.

**Table 3:** Sex differences in NPY Receptor Expression Levels.

Tissue	Experimental Treatment	Receptor Subtype	Result	Species	Reference
Cortex	None	Y1R	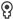 < 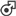 membrane protein levels 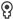 > 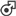 Y1R-NPY binding affinity 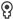 = 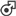 G_i_α levels	Wistar-Kyoto Rats	[72]
Locus Coeruleus	Single Prolonged Stress	Y1R	Stressed > unstressed mRNA levels ( 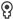 only)	Sprague Dawley Rats	[56,136,137]
Locus Coeruleus	Single Prolonged Stress	Y2R	Stressed < unstressed mRNA levels ( 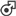 only)	Sprague Dawley Rats	[56,136,137]
